# Cross calibration of the Siemens mMR: easily acquired accurate PET phantom measurements, long term stability and reproducibility

**DOI:** 10.1186/2197-7364-2-S1-A28

**Published:** 2015-05-18

**Authors:** Sune H Keller, Bjorn Jakoby, Adam Espe Hansen, Susanne Svalling, Thomas L Klausen

**Affiliations:** Rigshospitalet, University of Copenhagen, Copenhagen, Denmark; University of Surrey, Guildford, UK

We present a quick and easy method to perform quantitatively accurate PET scans of typical water-filled PET plastic shell phantoms on the Siemens mMR PET/MR scanner. We perform regular cross calibrations (Xcals) of our PET scanners, including the Siemens mMR PET/MR, with a Siemens mCT water phantom. We evaluate the mMR cross calibration stability over a 3-year period. Recently, the mMR software (VB20P) offered the option of using predefined μ-maps. We evaluated this option by using either the predefined μ-map of the long mMR water phantom or a system-integrated user defined CT-based μ-map of the mCT water phantom used for Xcal. On 54 cross calibrations that were acquired over 3 years, the mMR on average underestimated the concentration by 16% due to the use of MR-based μ-maps. The mMR produced the narrowest range and lowest standard deviation of the Xcal ratios, implying it and is the most stable of the 6 scanners included in this study over a 3 year period. With correctly segmented μ-maps, the mMR produced Xcal ratios of 1.00-1.02, well within the acceptance range [0.95-1.05]. Measuring the concentration in a centrally placed cylindrical VOI allows for some robustness against misregistration of the μ-maps but it should be no more than a few millimeters in the x-y plane, while the tolerance is larger on the z-axis (when, as always with PET, keeping clear of the axial edges of the FOV). The mMR is the most stable scanner in this study and the mean underestimation is no longer an issue with the easily accessible μ-map, which in all 7 tests resulted in correct Xcal ratios. We will share the user defined μ-map of the mCT phantom and the protocol with interested mMR users.

